# Comparison of spike parameters from optically identified GABAergic and glutamatergic neurons in sparse cortical cultures

**DOI:** 10.3389/fncel.2014.00460

**Published:** 2015-01-14

**Authors:** Keiko Weir, Oriane Blanquie, Werner Kilb, Heiko J. Luhmann, Anne Sinning

**Affiliations:** Institute of Physiology, University Medical Center of the Johannes Gutenberg UniversityMainz, Germany

**Keywords:** neuronal culture, multi-electrode array, imaging, interneurons, network activity, spike waveform

## Abstract

Primary neuronal cultures share many typical features with the *in vivo* situation, including similarities in distinct electrical activity patterns and synaptic network interactions. Here, we use multi-electrode array (MEA) recordings from spontaneously active cultures of wildtype and glutamic acid decarboxylase 67 (GAD67)-green fluorescent protein (GFP) transgenic mice to evaluate which spike parameters differ between GABAergic interneurons and principal, putatively glutamatergic neurons. To analyze this question we combine MEA recordings with optical imaging in sparse cortical cultures to assign individual spikes to visually-identified single neurons. In our culture system, excitatory and inhibitory neurons are present at a similar ratio as described *in vivo*, and spike waveform characteristics and firing patterns are fully developed after 2 weeks *in vitro*. Spike amplitude, but not other spike waveform parameters, correlated with the distance between the recording electrode and the location of the assigned neuron’s soma. Cluster analysis of spike waveform properties revealed no particular cell population that may be assigned to putative inhibitory or excitatory neurons. Moreover, experiments in primary cultures from transgenic GAD67-GFP mice, which allow optical identification of GABAergic interneurons and thus unambiguous assignment of extracellular signals, did not reveal any significant difference in spike timing and spike waveform parameters between inhibitory and excitatory neurons. Despite of our detailed characterization of spike waveform and temporal spiking properties we could not identify an unequivocal electrical parameter to discriminate between individual excitatory and inhibitory neurons* in vitro*. Our data suggest that under *in vitro* conditions cellular classifications of single neurons on the basis of their extracellular firing properties should be treated with caution.

## Introduction

In contrast to intracellular or whole-cell patch-clamp recordings from individual neurons, where electrophysiologically studied cells can be correlated *post hoc* with morphological and immunohistochemical properties, the direct assignment of extracellularly recorded spikes to single neurons *in vivo* or in slice preparations is technically more challenging. However, only an unequivocal assignment of extracellularly recorded electrical signals to individual neurons allows detailed analyses of the spatio-temporal interactions in neuronal networks. *In vitro* cell cultures provide a useful model to study the spatio-temporal properties of single-cell firing with extracellular electrodes and developmental changes of these properties. Primary neurons in culture assemble to neuronal networks and develop spontaneous activity, which shares many features with early activity in developing brain structures *in vivo* and hence are commonly used to investigate principle mechanisms of neuronal network interactions (Kamioka et al., 1996; Voigt et al., 1997; Wagenaar et al., 2006; Baltz et al., 2010; Sun et al., 2010). Multielectrode arrays (MEAs) are a powerful and widely used method to record extracellular activity from large populations of neurons (Gross et al., 1982; Van Pelt et al., 2004; Chiappalone et al., 2006; Johnstone et al., 2010; Nimmervoll et al., 2013).

The read-out of most MEA-based studies largely profit from expanded analyses to single unit activity and cell-type specific assignment of neuronal signals. Combinations of spike waveform and spike timing parameters from intra- and extracellular recordings were successfully used to distinguish between GABAergic interneurons and excitatory neurons *in vivo* (Mountcastle et al., 1969; Csicsvari et al., 1999; Henze et al., 2000; Klausberger et al., 2003; Barthó et al., 2004; Courtin et al., 2014; Reyes-Puerta et al., 2014). Indirect cluster analysis of spike timing and spike waveform parameters suggests that an identification of interneurons in extracellular recordings may be feasible in neuronal cultures (Becchetti et al., 2012; Puia et al., 2012), but up to now a direct confirmation is missing. To provide a direct proof that extracellular spikes can be reliably assigned to distinct neuronal cell types on the basis of spike timing and waveform parameters, we used in the present study a combination of MEA recordings with optical imaging from sparsely cultured neurons, which enabled us to assign extracellular spikes to single, visually-identified neurons.

Extracellular spike waveforms depend critically on the maturational state of the neuron and the spatial orientation of a neuron relative to the recording electrode. Since cellular and network properties of cortical cultures undergo massive developmental alterations during the first weeks *in vitro* (Ichikawa et al., 1993; Kamioka et al., 1996; Boyer et al., 1998; Dabrowski et al., 2003; Sun et al., 2010), we first had to characterize developmental changes in spike waveforms in our sparse culture system. A direct influence of the neuron’s spatial orientation relative to the recording electrode on the recorded spike waveform has been previously suggested by modeling studies (Gold et al., 2006, 2007) and experimentally confirmed by high-density MEAs (Franke et al., 2012; Delgado Ruz and Schultz, 2014). We firstly had to investigate how the distance between the neuron and the recording electrode influences the recorded spike waveform properties in our MEA low-density culture system. After the assessment of spikes in a developmental and spatial context, we addressed the main question of this study, whether extracellular spike properties can be used to discriminate between inhibitory and excitatory cells. Therefore, we recorded spikes from cell sparse neocortical neuronal cultures generated from glutamic acid decarboxylase 67 (GAD67)-green fluorescent protein (GFP) transgenic mice that allow visual identification of GABAergic neurons.

Our experiments demonstrate (i) that the combination of extracellular spike recordings and optical imaging from sparsely cultured neurons on MEAs allows the unambiguous assignment of extracellular spikes to a single neuron; (ii) that in spite of a low density, cortical cultures develop normally and spike waveforms mature during the second week in culture; and (iii) that spike waveforms and discharge patterns are insufficient parameters to discriminate between excitatory principal and inhibitory GABAergic neurons *in vitro*. Parts of this study have been presented in abstract form (Weir et al., 2014).

## Materials and methods

### Cell culture

Neonatal mice, born and housed in the local animal facility, were used for this study. In some experiments, mice from the GAD67-GFP knock-in mice line were used, which are heterozygous for the expression of GFP in cells positive for GAD67-GFP (Tamamaki et al., 2003). All experiments were conducted in accordance with national and European (86/609/EEC) laws for the use of animals in research and were approved by the local ethical committee. In brief, newborn mice were decapitated at postnatal day (P) 0–1 and brains were transferred to ice-cold Ca^2+^ and Mg^2+^ free HBSS (Gibco, Invitrogen, Carlsbad, CA, USA) supplemented with penicillin and streptomycin (50 units/ml) and 10 mM HEPES. From the cerebral hemispheres, meninges were removed, and the neocortex was isolated from the hippocampus, striatum and thalamic nuclei. Tissue was incubated in modified HBSS with 0.05% trypsin/EDTA at a temperature of 37°C for 20 min followed by DNase digestion (5 min, room temperature). Mechanical dissociation of neocortical cells was performed by repetitive titration through fire-polished pipettes with declining diameter. Trypsinization was blocked by rinsing cells with HBSS followed by Minimal Essential Medium (Gibco) supplemented with 10% horse serum and 0.6% glucose. Cells were counted in a Neubauer chamber and seeded at two different cell densities. For the medium sparse cultures 1500 cells were plated per mm^2^ and for the sparse cultures 900 cells were plated initially per mm^2^. Cells were plated on MEAs which were coated with polyethyleneimine (Sigma, 0.05% in Borat-buffered solution) at RT for 45 min prior to plating. For immunohistochemical staining, neurons were plated on polyornithin-coated coverslips. Culture medium was completely exchanged to a culture medium consisting of Neurobasal medium (Gibco) supplemented with 2% B27 (Gibco), and 1 mM L-glutamine after 1 h. Cells were cultivated at 37°C and 5% CO_2_. One-third of the culture medium was exchanged every 7 days excluding the week of recording.

### Immunocytochemistry

For immunohistochemical analysis, cells were fixed in 4% PFA in PBS for 15 min after 14 days *in vitro* (DIV). Overnight stainings were performed with the following primary antibodies: monoclonal mouse anti Somatostatin (Biozol), NeuN (Millipore) and GAD67 (Millipore), polyclonal rabbit anti Parvalbumin (Swant) and NeuN (Millipore) as well as Cy3 and DyLight488 coupled secondary antibodies (Dianova and Biomol; 2 h at RT). Images were taken with 20× and 40× objectives with an Olympus IX81 epifluorescence microscope and subsequently analyzed with ImageJ.

### Electrophysiology

Cell cultures were established on MEAs containing 120 planar extracellular titanium nitrite electrodes with four internal references (120MEA100/30iR-Ti-gr, Multi Channel Systems, Reutlingen, Germany). MEAs had an electrode diameter of 30 μm and an interelectrode spacing of 100 μm. Signals from 120 recording electrodes were recorded with MC_RACK software (Multi Channel Systems) in a MEA 2100 system (Multi Channel Systems) at a sampling rate of 50 kHz and high-pass filtered at 200 Hz. Cultures were recorded after 14–16 DIV. A subset of recordings was performed after 7–8 DIV and 21–22 DIV. Spikes were detected using a threshold-based detector set to a threshold of 7× the SD of the noise level (MC_RACK, Multi Channel Systems). All electrophysiological recordings were performed in artificial cerebrospinal fluid (aCSF) that resembled the culture medium (Cohen et al., 2008) (in mM): 129 NaCl, 5.3 KCl, 2 CaCl_2_, 1 MgCl_2_, 10 D(+)-glucose, 26 NaHCO_3_ and gassed with 95% O_2_/5% CO_2_. ACSF was perfused at a rate of 1 ml/min and temperature was maintained at 32°C by a temperature controller (TC02, Multi Channel Systems). Spike datasets from all electrodes were imported into Matlab 7.7 (Mathworks, Natick, MA, USA) for analysis using a custom written routine.

### Spike sorting and analysis

Spike sorting was carried out as described previously (Sun et al., 2010). In brief, spike sorting was performed using k-means algorithm based cluster analysis, with the number of clusters estimated by visual inspection. The spike datasets consisted of vectors for all neurons.

Autocorrelation functions were applied to confirm spike sorting and cross-talk between electrodes was excluded by analysis of correlation coefficient matrices for analyzed MEAs (*n* = 516 neurons from *n* = 7 medium density and *n* = 11 low density cultures).

**Figure 1:**
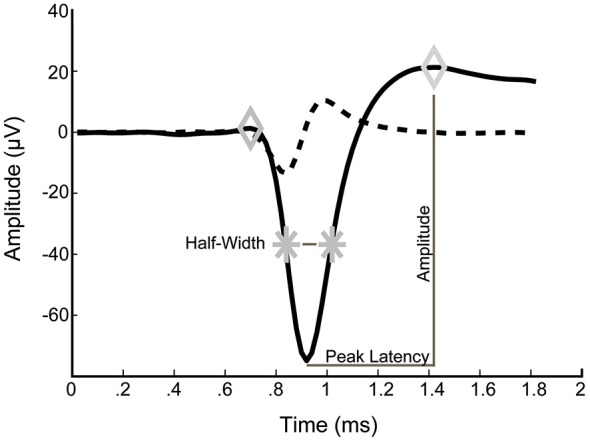
**Typical example of an averaged spike waveform from a 14 DIV culture**. Left and right diamonds correspond to detected maxima. Stars indicate spike half-amplitude. Dashed line is the first derivative of the average waveform.

For analysis of spike waveforms, the voltage amplitude, peak latency, spike half-width and asymmetry were determined for each averaged spike (Figure 1). Average spike waveforms were calculated over a recording period of 10 min and only neurons with at least 10 spikes were included. Voltage amplitude was defined as the voltage difference between the second maximum and the minimum. Peak latency was defined as the time between the minimum and the second maximum. Spike half-width was calculated as the width of the spike at half-maximal amplitude of the first maximum. Asymmetry was defined as the ratio of the amplitude of the second maximum to that amplitude of the first maximum.

Average firing frequencies were calculated as arithmetic mean of individual firing frequencies of all identified units. A burst was defined as three or more spikes with an interspike interval (ISI) less than 50 ms (Wagenaar et al., 2006). Based on this we determined the burst duration (BD), mean interburst interval (IBI) and mean number of spikes in a burst for each neuron. Additionally, ISI within a 800 ms time interval were calculated for each neuron and burst ISI ratio (number of spikes with ISI <100 ms/number of spikes with ISI ≥100 ms) (Viskontas et al., 2007). The burst index (BI) was calculated as described before (Wagenaar et al., 2005; Sun et al., 2010). Finally, the Fano Factor for ISIs less than 800 ms and the coefficient of variation (CV) of the ISI were analyzed. An overview of all analyzed spike waveform and spiking parameters is given in Table 1.

**Table 1:** **Properties of GFP-negative and GFP-positive neurons**.

	GFP-negative neurons	GFP-positive neurons	Significance (Mann–Whitney Test)
*Spike waveform properties*
Amplitude (μV)	87.09 ± 8.35	54.10 ± 8.74	*p* = 0.01
Asymmetry	1.14 ± 0.03	1.13 ± 0.06	*p* = 0.89
Spike width (ms)	0.28 ± 0.01	0.23 ± 0.02	*p* = 0.07
Trough to Peak (ms)	0.69 ± 0.02	0.55 ± 0.04	*p* = 0.11
*Spike temporal properties*
Firing frequency (Hz)	0.26 ± 0.04	0.44 ± 0.11	*p* = 0.18
Intraburst firing frequency (Hz)	54.83 ± 24.25	54.58 ± 11.73	*p* = 0.30
Mean IBI (s)	75.85 ± 16.02	64.49 ± 29.58	*p* = 0.37
Average burst duration (s)	0.08 ± 0.003	0.11 ± 0.03	*p* = 0.60
Average spikes per burst	3.63 ± 0.11	5.679 ± 1.694	*p* = 0.29
Mean ISI (ms)	187.40 ± 14.44	224.70 ± 41.69	*p* = 0.74
Variance ISI	28953 ± 4552	26175 ± 5195	*p* = 0.63
CV ISI	0.79 ± 0.03	0.94 ± 0.12	*p* = 0.37
CV ISI^2^	0.69 ± 0.047	1.12 ± 0.27	*p* = 0.37
ISI ratio	0.65 ± 0.10	0.77 ± 0.33	*p* = 0.58
Fano Factor	2.83 ± 0.30	3.23 ± 1.03	*p* = 0.48

### Imaging

Imaging of neuronal activity on MEAs was performed with an upright microscope (Olympus BX61WI with a Hamamatsu Orca R^2^ C10600 CCD camera) to ensure that no cells were masked by an electrode. For cell body imaging, cells were stained with 1 μM calcein red-orange (Life Technologies, Eugene, OR, USA) with 0.02% pluronic acid in 1 ml aCSF for 10 min. Fluorescence was recorded with a 10× water-immersion objective and a red filter set (excitation 560/20 nm, emission 607/25 nm). For calcium imaging, cells were stained with 10 μg/ml of Oregon Green 488 (Life Technologies, Eugene, OR, USA) with 0.02% pluronic acid in 1 ml of aCSF for 20 min. Fluorescence was recorded with a 10× water-immersion objective at a frequency of 2 Hz and a MT20 light source (Olympus) with a green filter set (excitation 470/20, emission 525/25 nm). For calcium imaging, regions of interests (ROI) were manually set to the soma of the cell. ΔF plot was calculated based on the change in fluorescence from baseline and bleach corrected. For a subset of neurons, percentage changes in fluorescence during bursts were correlated with the number of spikes per burst.

Images were tiled, aligned and merged in ImageJ using MosaicJ (Thévenaz and Unser, 2007). Cell bodies and electrodes were detected using a custom image analysis routine in Matlab. All images were adjusted for brightness and contrast and converted to black and white. ROIs were identified using an edge detection routine.

### Statistics

Values are given as mean values ± S.E.M. All statistical tests were performed using Prism 5 (GraphPad, La Jolla, CA, USA) or appropriate Matlab functions. Comparisons between age groups and culture groups were performed with a one-way ANOVA and a Tukey’s Multiple Comparison Test for *post hoc* analysis. Comparisons between two groups were performed with Students unpaired *T*-test or Mann–Whitney test when applicable. Significance was considered at *p* values <0.05.

## Results

**Figure 2:**
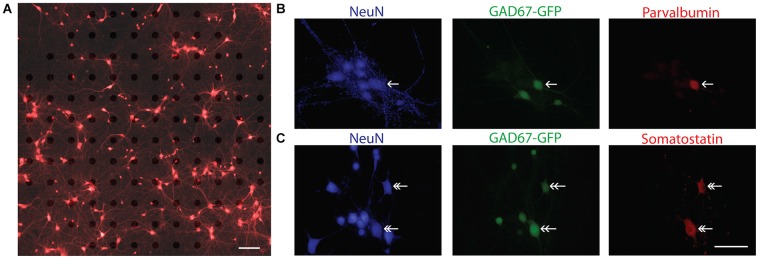
**Characterization of sparse cortical cultures. (A)** Overlay of brightfield and fluorescence images of calcein red-orange stained cells sparsely cultured on a 120-channel MEA recorded at 14 DIV. Note, electrodes are visible in brightfield image. In this example, 186 neurons were detected in the field of view by our imaging analysis and 13 unique spike waveforms from 12 electrodes were detected. Scale bar is 100 μm. **(B,C)** Immunocytochemistry confirms the presence of parvalbumin positive (single arrow head) and somatostatin positive (double arrow head) neurons. In **(B)**, NeuN (blue), GAD67-GFP (green), parvalbumin (red). In **(C)**, NeuN (blue), GAD67-GFP (green), somatostatin (red). Scale bar is 50 μm.

For the present study, a total of 28 cultures from 15 independent preparations of wild type (WT) and heterozygous GAD67-GFP mice at two different plating densities were analyzed. In order to visually confirm the cellular source of an electrical signal recorded on a MEA, cortical neurons were plated at the lowest possible density achieved with consistent cell culture survival for 3 weeks. A typical low density culture after 14 DIV is illustrated in Figure 2A. In these cultures the mean density at DIV 14 was 115.2 ± 19.9 neurons/mm^2^ (*n* = 8 cultures). To ensure that GABAergic neurons were present in these cultures at a ratio similar to that *in vivo*, WT cultures were fixed at DIV 14 and immunostained for the neuronal marker NeuN and GAD67, an isoform of the synthetic enzyme GAD. In three cultures 16.05 ± 1.92% of neurons (*n* = 24 fields of view, six coverslips) were positively labeled for GAD67. The presence of different interneuron subtypes was confirmed by immunohistochemical analysis of WT and GAD67-GFP cultures with antibodies against parvalbumin and somatostatin (Figures 2B,C).

### Spike waveforms and cortical networks mature within first 2 weeks *in vitro*

Although a variety of previous MEA studies investigated the development of network activity patterns during the first weeks *in vitro* (Wagenaar et al., 2006; Sun et al., 2010), information on the development of spike waveform properties is missing. Therefore we analyzed the properties of spontaneous activity patterns between DIV7 and DIV22 in 1 week intervals with special emphasis on the development of extracellular spike waveform properties.

In accordance with previous reports (Wagenaar et al., 2006; Sun et al., 2010) firing frequency and bursting parameters mature over the course of the first 3 weeks in culture. Cultures at DIV 7/8 showed activity in few cells (11.75 ± 2.25 neurons, *n* = 41 neurons/4 cultures) and at a low firing frequency (0.18 ± 0.05 Hz). Although activity was already synchronized at DIV 7/8 (BI 0.74 ± 0.14, *n* = 4 cultures), bursts could only be detected infrequently, in 8 out of 41 neurons. After 2 weeks in culture average firing frequency significantly increased (0.48 ± 0.04 Hz, *n* = 514 neurons, 18 cultures, one-way ANOVA, *F* = 5.31 *p* = 0.0051, Tukey’s *p* < 0.01) and activity was mostly synchronized occurring within bursts (BI 0.74 ± 0.04, intra-burst firing frequency 52.99 ± 0.92 Hz, *n* = 236 neurons/18 cultures). No major changes in firing frequency or bursting parameters could be observed at later time points. At DIV 21/22 activity was detected at a firing frequency of 0.39 ± 0.05 Hz (*n* = 93 neurons in 4 cultures, Tukey’s *p* > 0.05) with a BI of 0.87 ± 0.07 (*T*-test *p* = 0.20). Intra-burst firing frequency was slightly increased to 54.97 ± 11.3 (*n* = 53 neurons in 4 cultures, Mann–Whitney test *p* < 0.001).

**Figure 3:**
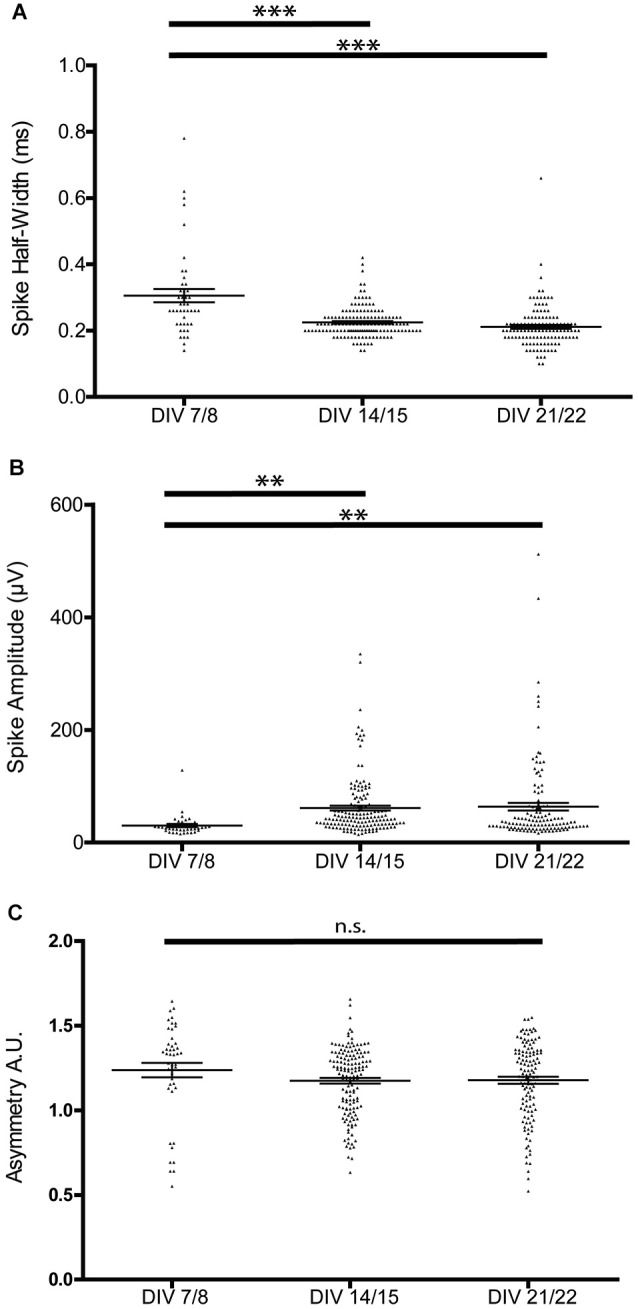
**Developmental changes in spike waveform properties. (A)** Spike half-width narrows during the first 2 weeks of culture, but is not significantly different between the second and third week of culture. **(B)** Spike amplitude increases during the first 2 weeks of culture, but is not significantly different between the second and third week of culture (one-way ANOVA, **p < 0.01 ***p < 0.005). **(C)** Spike asymmetry is not significantly different between the recorded stages.

This stabilization of the bursting patterns around P14 was paralleled by the maturation of extracellular spike parameters. Spike half-width decreased during the first 2 weeks in culture (Figure 3A), but was not significantly different between 2 and 3 weeks in culture (7/8 DIV 0.31 ± 0.02 ms, 14/15 DIV 0.23 ± 0.004 ms, 21/22 DIV 0.21 ± 0.01 ms, *n* = 44/145/119, one-way ANOVA, *F* = 28.45 *p* < 0.0001). As expected, the width of spikes positively correlated with the calculated trough-to-peak time (*n* = 78, *R*^2^ = 0.33, *p* < 0.001).

Spike amplitudes increased significantly during the first 2 weeks in culture and were again comparable between the 2nd and 3rd week in culture (7/8 DIV 30.14 ± 2.75 μV, 14/15 DIV 61.26 ± 4.35 μV, 21/22 DIV 63.70 ± 6.83 μV, *n* = 42/150/122, one-way ANOVA *F* = 5.27 *p* = 0.006; Figure 3B). The spike asymmetry did not change over the analyzed culture period (7/8 DIV 1.24 ± 0.04, 14/15 DIV 1.17 ± 0.02, 21/22 DIV 1.18 ± 0.02, *n* = 45/147/122, one-way ANOVA, *F* = 1.47 *p* > 0.05; Figure 3C).

In summary, these results indicate that single cell and neuronal network activity, but also the features of single unit spikes, showed mature characteristics at the end of the second week in culture. Therefore, we performed further analyses at this developmental stage.

### Extracellular activity can be assigned to individual optically identified cortical neurons in sparse cortical cultures

Extracellular spikes recorded on widely spaced MEAs can only be assigned to single neurons in low density cultures. Therefore, cortical neurons were cultured at low plating densities. In cultures with two different plating densities, medium sparse and sparse, no major differences in electrical activity patterns were detected after 2 weeks in culture. On average, electrical activity in sparse cortical cultures at 14–15 DIV was recorded from 17.7 ± 1.6 neurons (*n* = 371 neurons, 21 cultures) with an average firing frequency of 0.33 ± 0.03 Hz. In medium sparse cultures, unique spike waveforms were detected from 46.9 ± 3.9 neurons (*n* = 328 neurons, 7 cultures) and spikes occurred at a significantly higher firing frequency of 0.54 ± 0.05 Hz (Mann–Whitney test, *p* = 0.01). Both types of cultures exhibited similar bursting patterns, characterized by periods of silence and synchronized bursting. This was quantified by the BI, which was 0.70 ± 0.05 for low density cultures and 0.71 ± 0.06 for medium sparse cultures (*T*-test, *p* = 0.93) and the intra-burst firing frequency (medium sparse cultures 54.96 ± 2.00 Hz, sparse cultures 53.23 ± 1.02 Hz, *n* = 178/201 neurons, Mann–Whitney test, *p* = 0.17).

**Figure 4:**
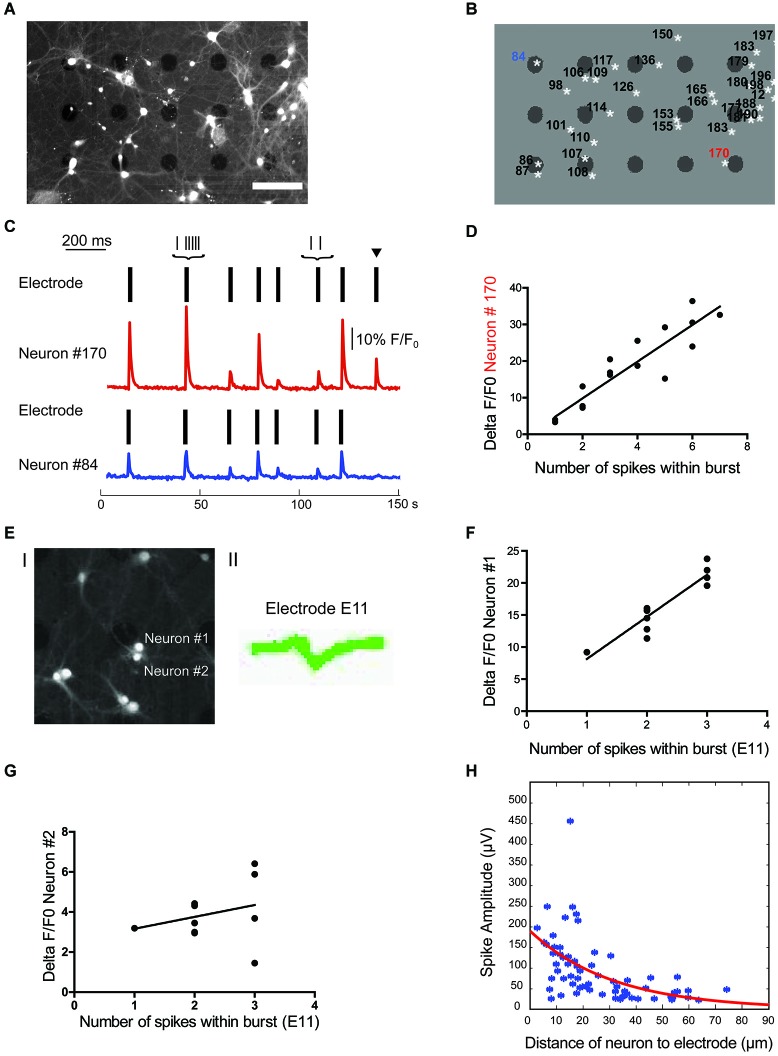
**Localization of cell soma correlates with spike waveform amplitude. (A)** Overlay of brightfield and fluorescence image of cells from a culture at 14 DIV. Scale bar is 100 μm. **(B)** Regions of interest (ROIs) for cell bodies and electrode locations were automatically detected in Matlab. White stars indicate the center of mass of cell ROIs, black dots indicate electrodes. **(C)** Source of electrical activity recorded at a single electrode can be confirmed with calcium imaging. Spike times recorded from two separate electrodes are each indicated by a rasterplot. A F/F_0_ plot from the neurons closest to the active electrodes in example B is also included. Note, black arrow marks an event that can only be detected on electrode close to neuron #170. Scale bar is 10% F/F_0_ and 200 ms, respectively. **(D)** Number of spikes within a burst is linearly correlated with F/F_0_ (*R*^2^ = 0.748 for neuron #170). **(E)** Fluorescence signal of cells close to one electrode (#E11) after staining with calcium indicator merged with a bright field image to localize electrode E11 (I) as well as the single cluster of spike waveforms detected at electrode E11 (II). **(F,G)** Correlation of spikes detected at electrode E11 with relative change in calcium signal of nearby neuron #1 **(F)** and neuron #2 **(G)**. Only calcium signal from neuron #1 correlates significantly with number of spikes within a burst detected electrically (neuron #1: *R*^2^ = 0.859 and *p* < 0.0001; neuron #2: *R*^2^ = 0.076 and *p* = 0.41). **(H)** Spike amplitude decays exponentially with distance between the cell soma and electrode (*n* = 66, *R*^2^ = 0.279).

In order to be able to definitely assign extracellular spikes to single neurons we combined electrophysiological recordings of sparse cultures with simultaneous imaging. This approach allowed us to analyze cell body positions relative to electrode positions using a custom Matlab analysis routine. Here, ROIs were identified corresponding to the center of the soma of calcein-stained neurons in fluorescent images and electrodes in brightfield images. An example of this imaging analysis is shown in Figure 4. In low density cultures, active electrodes typically only detected spikes from a single neuron, as determined by our spike sorting analysis. In sparse wild-type cultures recorded after 14 DIV, single neuron activity was detected by 81.6% of all active electrodes, electrical activity from two neurons was detected at 16.8% of all electrodes and only a very few electrodes (1.6%) picked up a signal from more than two neurons.

Upon visual inspection of cell body position relative to the position of electrodes, it became evident that in many instances where only one spike waveform was detected on an active electrode, this electrode was touching or clearly in close proximity to a single neuron. An example of this signal-source identification is shown in Figures 4B,C. Here, electrodes close to neurons labeled as ROI #84 and #170 both detected spikes from a single neuron and allowed a clear assignment of the spikes to one neuron close to the active electrodes. This assignment was confirmed by simultaneously recording spiking activity on electrodes and calcium imaging at the soma of the nearby neuron. Although spikes mostly occur in synchrony, rare non-synchronous spikes allowed the definite assignment of relative changes in calcium of neurons to detected spikes on nearby electrodes. Accordingly, a single calcium signal within the soma of the nearby neuron was always correlated with at least a single spike detected electrically and we failed to observe fast calcium transients in all electrically silent cells close to the electrode (e.g., ROI 153 and ROI 155 in Figures 4A,B). Further, the number of spikes within a burst detected at the electrode was positively correlated with the concurrent percentage increase in fluorescence within the assigned neuron. As illustrated in Figure 4D for a single neuron, the number of spikes within a burst correlated with the percentage change in fluorescence (*R*^2^ = 0.67 ± 0.08, *n* = 6 neurons from 3 cultures). This approach of combining extracellular recordings with calcium imaging would, in principle, also allow the assignment of spikes to single neurons even if several neurons are in close proximity to the same electrode. As a proof of principle we analyzed the correlation of relative changes in intracellular calcium of multiple, nearby neurons with the number of spikes detected with one electrode. As shown in Figures 4E–G, we could always detect a positive correlation for one of the close-by neurons (*R*^2^ = 0.461/0.859/0.507 and *p* = 0.0051/0.0001/0.014) and the absence of such in the remainder (*R*^2^ = 0.025/0.076/0.023 and *p* = 5.57/0.41/0.13; *n* = 3).

However, in cases where the source of a signal could not be clearly identified, i.e., when several neurons were in proximity of an electrode (e.g., ROI 86 and ROI 87 in Figures 4A,B) and/or more than one unique spike waveform was detected by an electrode (e.g., electrode close to ROI 183 and ROI 179 in Figures 4A,B), data were excluded from further analysis.

In summary, these results confirm that our sparse culture system allows us to confidently identify the source of the electrical signals.

### Spatial orientation of recorded neuron affects spike waveform

Theoretical considerations from modeling studies (Gold et al., 2007; Pettersen and Einevoll, 2008) as well as experimental data from *in vivo* (Buzsáki, 2004) and *in vitro* high-density MEA recordings (Franke et al., 2012; Delgado Ruz and Schultz, 2014) suggest that the spatial relationship between the neuron and the recording electrode has a strong influence on the recorded spike properties. To address this question we correlated this distance between the center of the active electrode and the center of the assigned neuronal soma with the following spike waveform parameters from the electrical recordings: amplitude, half-width, asymmetry, and trough-to-peak time. Spike amplitude for visually confirmed active neurons exponentially decayed with distance to electrode (Figure 4H, *R*^2^ = 0.28, *n* = 66). It is important to note that a few neurons were detected further than 50 μm from the center of mass of the neuron’s ROI, which is more than half of the inter-electrode distance. However, as described in the methods, we could not find an example of two neighboring electrodes recording from the same neuron. On the other hand spike half-width, asymmetry and trough-to-peak time were not linearly correlated with distance (*R*^2^ = 0.02, 0.02 and 0.07 respectively, *n* = 66). From these results we conclude that the only spike waveform parameter that correlated with the neuron-to-electrode distance is the spike amplitude. Thus, location of the recorded neuron will only have a minor impact on spike waveform.

### GABAergic interneurons cannot be identified *in vitro* based on spike properties

After we established that the spike-waveform reach mature levels at 14 DIV and are not influenced by the spatial relationship between the recorded neurons and the electrode, we tried to use the spike waveform parameters to distinguish between putative excitatory and inhibitory neurons (Csicsvari et al., 1999; Barthó et al., 2004; Viskontas et al., 2007; Sakata and Harris, 2009; Buetfering et al., 2014; Reyes-Puerta et al., 2014). However, in constrast to these *in vivo* studies, we were not able to classify two divergent populations of neurons on basis of the elecrophysiological parameters spike half-width, spike asymmetry or spike latency in recordings from sparse WT cultures (Weir et al., 2014), although these cultures clearly contained GABAergic neurons (see Figure 2). These observations demonstrate that putative inhibitory neurons cannot be discriminated from excitatory ones by spike properties *in vitro* using classical spike waveform parameters.

In order to provide further evidence for this proposal and to reveal other distinguishing features between GABAergic and non-GABAergic neurons, we next investigated sparse cortical cultures from GAD67-GFP knock-in mice. In total 10 GAD67-GFP cultures were recorded after 14–15 DIV and spikes from 151 neurons were analyzed.

No significant differences were observed between spike timing properties of sparse cultures from WT mice compared to sparse cultures from GAD67-GFP mice at DIV 14–15 (firing frequency: WT 0.37 ± 0.04 Hz, GAD67 0.30 ± 0.03 Hz, *n* = 187/184, *p* = 0.12; BI: WT 0.76 ± 0.06, GAD67 0.64 ± 0.09, *n* = 11/10 cultures, *p* = 0.34; intra-burst firing rate: WT 0.40 ± 0.07, GAD67 0.32 ± 0.04, *n* = 86/92 neurons, *p* = 0.52).

**Figure 5:**
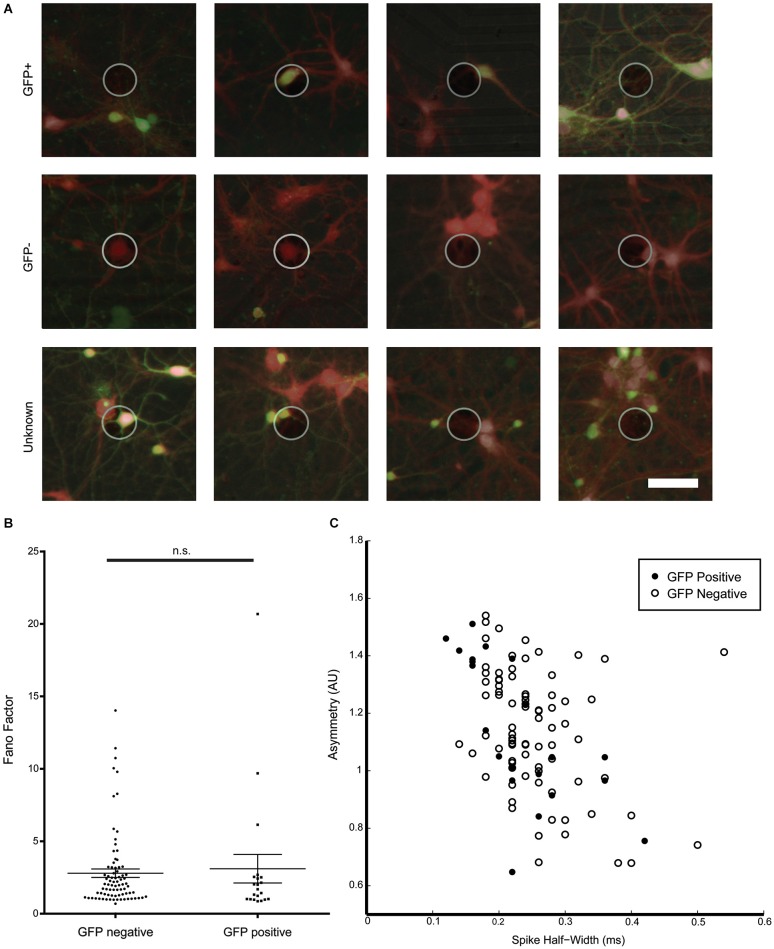
**Characterization of spike waveforms recorded from electrodes with GFP-positive and GFP-negative neurons. (A)** Example images of active electrodes from a GAD67-GFP culture. Brightfield, GFP (green), and consecutive calcein red-orange (red) images were manually overlaid. Top row: spikes assigned a GFP-positive neuron. Middle row: spikes assigned to a GFP-negative neuron. Bottom row: spikes cannot be clearly assigned to GFP-positive or GFP-negative neurons. **(B)** No significant difference between Fano Factors for GFP-positive and GFP-negative neurons. **(C)** Cluster analysis of spike asymmetry vs. half-width. Filled circles are GFP-positive neurons, open circles are GFP-negative neurons.

For most electrodes, activity was recorded from a single neuron (78.8%) and only in rare instances, two (20.5%) or three neurons (0.7%) were detected on one electrode. This low density culture allowed us to visually confirm the presence or absence of a GFP-signal in the neuron assigned as the source of a signal detected on a nearby electrode and allowed us to compare spike waveform properties between signals recorded from GFP-positive GABAergic as well as from GFP-negative non-GABAergic neurons. Figure 5A shows example images of active electrodes with brightfield, calcein, and GFP images superimposed. In some cases, multiple neurons were in close proximity to an active electrode. In cases where a group of neurons near an active electrode were homogeneously GFP-positive or GFP-negative, the recorded spikes could still be assigned to GFP-positive or GFP-negative neurons, respectively, otherwise the signals were rejected.

Spike waveform properties as well as spike timing properties were analyzed for 21 identified GFP-positive and 82 GFP-negative neurons in an effort to find distinguishing characteristics for the two populations. A total of 16 parameters were analyzed and compared between GFP-positive and GFP-negative neurons. As shown in Table 1, no significant difference between groups was found in any of the analyzed spike timing and waveform parameters, except for the amplitude of the averaged spike waveform. Spikes assigned to GFP-negative neurons were significantly smaller (54.1 ± 8.7 μV, *n* = 21) compared to spikes detected from GFP-positive neurons (87.1 ± 8.4 μV, *n* = 82, Mann–Whitney test, *p* = 0.01). In addition, most of these parameters showed a high variance. In particular the kinetics of the spikes, represented by spike width and through-to-peak time, have been used to discriminate between putative GABAergic and glutamatergic neurons (Csicsvari et al., 1999; Barthó et al., 2004; Sakata and Harris, 2009; Reyes-Puerta et al., 2014). But neither one of the two parameters was significantly different between spikes from GFP-positive and GFP-negative neurons *in vitro* (Table 1, Spike width: 0.23 ± 0.02 ms, 0.28 ± 0.01 ms, *n* = 20/81, Mann–Whitney test *p* = 0.067; trough-to-peak: 0.69 ± 0.02 ms, 0.55 ± 0.04 ms, Mann–Whitney test *p* = 0.11). It should be noted that 43% of spikes assigned to GFP-positive neurons had a spike width less than 0.2 ms, while only 23% of spikes to GFP-negative neurons had a spike width less than 0.2 ms (compare to Figure 5C). While the mean values were not significantly different, the higher percentage of GFP-positive neurons with spikes less than 0.2 ms suggests that there is a tendency for GFP-positive neurons to have narrower spikes.

Also, none of the analyzed spike timing parameters were statistically different between GFP-positive and GFP-negative neurons. Figure 5B for example, shows Fano Factor values calculated for all identified GFP-positive and GFP-negative neurons. Statistically insignificant different means of both groups (Table 1), as well as overlapping values (Figure 5B) indicate that this could not be used to distinguish the two populations (GFP-positive 3.23 ± 1.03, GFP-negative 2.83 ± 0.30, *n* = 20/81, Mann–Whitney test, *p* > 0.05).

We next attempted an indirect 2D cluster analysis by plotting combinations of spike timing and waveform parameters to determine if populations can be distinguished by multiple variables. However, no combination of two variables resulted in clearly separated clusters that could distinguish the GFP-positive and GFP-negative neurons. Figure 5C shows for spike assigned to GFP-positive or GFP-negative neurons a cluster plot for asymmetry and spike-width, which is commonly used to distinguish between excitatory and inhibitory neurons *in vivo* (Csicsvari et al., 1999; Barthó et al., 2004; Sakata and Harris, 2009; Reyes-Puerta et al., 2014), These results support that spike waveform and spike timing properties were insufficient to distinguish GABAergic from non GABAergic neurons from their electrophysiological properties in sparse dissociated cell cultures.

The same analysis of spike timing and waveform parameters was also applied to medium density WT cultures (*n* = 328 neurons from 7 cultures) and yielded a similar variance of analyzed spike waveform and timing parameters. Also, neurons in medium dense cultures showed no clear clusters when combining different parameters in a cluster plot analysis.

In summary, these analyses revealed that it was not possible to discriminate extracellular spikes of inhibitory interneurons from those recorded from excitatory neurons *in vitro*, although we could reliably confirm their molecular identity on a single cell level.

## Discussion

The direct assignment of extracellular electrophysiological signals to single cortical neurons in the present study allowed us to assess the influence of spatial, developmental and cell-type dependent factors on spike waveform and spike timing parameters on a single cell and population based level. Recordings at different developmental stages confirmed that our cultures reach a stable mature stage after 2 weeks in culture with no further change in amplitude and widths of recorded spikes. Correlations of electrode to cell soma distance revealed a strong negative correlation of spike amplitude with distance to the recording electrode, whereas other parameters of spike waveform did not show such a correlation. Despite our effort to analyze 15 different spike waveform and spike timing parameters we neither found a single parameter nor a combination of such parameters that allowed us to reliably differentiate GABAergic interneurons from non-GABAergic neurons based solely on their electrophysiological signature *in vitro*.

For our experiments, extracellular activity was recorded from cultures at three different developmental time points in order to evaluate the developmental profile in our culture conditions and to choose a recording time point in which spike timing and waveform properties reach a stable plateau. At 7 DIV, cultures exhibited a small number of low amplitude and wide spikes, likely due to immature ion channel currents (Moody and Bosma, 2005). By 14 DIV however, cultures exhibited higher amplitude, narrower spikes and spike patterns were dominated by synchronized bursting events (Wagenaar et al., 2006; Sun et al., 2010; Weir et al., 2014). Low density cultures from WT and GAD67-GFP mice exhibited spontaneous activity and firing patterns at 14 DIV that were rather similar to higher density cultures (Sun et al., 2010; Biffi et al., 2013). At this stage, spikes had the same average amplitude and width as spikes recorded at 21 DIV, indicating that cultures reached a mature, stable state after 2 weeks. The increase in spike amplitude, as well as narrowing of the spikes during the first 2 weeks in culture, can be attributed to an increase in cellular size (Gold et al., 2007) as well as maturation of ion channel properties (Beckh et al., 1989; Moody and Bosma, 2005; Okaty et al., 2009).

The large variance in measured spike waveform parameters was unexpected in light of previous studies that have used such parameters to identify distinct populations of neurons (Csicsvari et al., 1999; Barthó et al., 2004; Viskontas et al., 2007; Sakata and Harris, 2009; Buetfering et al., 2014; Reyes-Puerta et al., 2014). We attribute this heterogeneity of spike waveforms to differences in developmental profile of cultured neurons compared to neurons *in vivo* (Dabrowski et al., 2003) as well as the random spatial distribution of the neurons relative to the recording electrode (Gold et al., 2006). The extracellular waveform, while not a direct reflection of the intracellular action potential, is strongly correlated to the shape of the spike recorded intracellularly (Henze et al., 2000). Yet, ion channel densities also vary within different subcellular compartments of a neuron, therefore we expect that the location of the soma relative to the recoding electrode will theoretically impact spike waveform parameters (Gold et al., 2006; Bauer et al., 2013). In recordings of similarly aged cultures on MEAs with smaller inter electrode distances, it is evident that signals originating from a single neuron recorded by multiple electrodes from different regions of the neuron highly varied in their spike waveforms (Franke et al., 2012). The combination of optical imaging using an upright microscope with MEA-based electrophysiological recordings of low density cultures allowed us to reliably match the extracellular signal to single neurons as confirmed by simultaneous calcium imaging. Simultaneous calcium imaging supports the correlation between localization of an active cell and the corresponding electrical signal. The tight correlation between the number of spikes and the amplitude of the calcium transient also clearly proves the identity between visually and electrically identified cells. A combination of fast calcium imaging and MEA recordings can in principle also allow the assignment of spikes to single neurons when multiple neurons are in close proximity of the electrode. Spike width or asymmetry did not correlate with the soma to electrode distance. Only the amplitude of the recorded spikes had a strong negative correlation with distance between the recording electrode and the assigned neuron, a result supported by previous extracellular recordings and modeling experiments (Henze et al., 2000; Gold et al., 2006, 2007; Pettersen and Einevoll, 2008). However, our distance calculation does not take into account differences in cell body size. Larger neurons are more likely to have part of their cell body touching an electrode than smaller neurons. As a result, larger neurons are more likely to have their measured centroids appear farther away than smaller neurons, and yet have a larger amplitude signal. Hence, we assume that we have a technical bias of approximately +/− 10 μm. To our surprise, when using 100 μm inter-electrode spaced 120 channel MEAs with 30 μm diameter electrodes, we found no instances of a neuron being detected by two electrodes simultaneously. This was confirmed by spike time correlation analyses and cross-correlation plots for neighboring active electrodes. There are likely multiple reasons to account for our lack of crosstalk. The first is our high spike detection threshold. Approximately 85% of source neurons identified were within 50 μm of the recording electrode (half of the inter-electrode spacing). The remaining 15%, while seemingly large, is due to a bias toward selecting and analyzing electrodes at the outer edge of the array. In these cases, it was also clear that the soma of the neuron was asymmetric and it was likely that the neuron’s axon extended toward the active electrode. The extracellular recording distance calculated from the present study *in vitro* is in agreement with previous results from *in vivo* recordings (Henze et al., 2000; Delgado Ruz and Schultz, 2014).

Out of the approximately 160 cells cultured in the recording field, signals from an average of 17.7 neurons were detected electrically in the low density cultures. This low detection rate of approximately 10% can be attributed to multiple causes: high threshold for spike detection, a low probability of a cell body touching an electrode and the presence of silent neurons and non-spiking cells in our mixed culture system. Indeed we observed neurons in close approximate to electrodes that did not generate detectable spikes. As previously discussed, our high threshold for spike detection reduced the probability of falsely attributing noise as a spike, but also reduced the number of spikes detected. Our analysis of distance and amplitude indeed showed that the center of the soma of most of the recorded neurons was located within 20 μm from the center of the recording electrode, indicating that most neurons need to be directly on top or touching the electrode to be detected electrically. The recording electrodes for the MEAs used in these experiments also only covered 5.9% of the total recordable area of the MEA. Thus the probability of a cell body touching an electrode was very low.

We attempted to use both waveform characteristics and spike timing properties to distinguish GABAergic interneurons in our cortical cultures. We ensured that these cultures consisted of a similar ratio of interneurons as described in the cerebral cortex *in vivo* (Markram et al., 2004) and that interneurons expressed typical interneuron markers, i.e., somatostatin and parvalbumin. Our rigorous identification ensured that spike waveforms were correctly assigned to GFP-positive or GFP-negative neurons. Differences in amplitude of recorded spikes between GABAergic and non-GABAergic neurons can most likely be attributed to known differences in somatic size (Hornung and De Tribolet, 1994). Unexpectedly, none of the additional 15 analyzed spike timing and spike waveform parameters showed significant differences between signals recorded from GFP-positive or -negative neurons. Spike widths assigned to GFP-positive and -negative neurons overlapped extensively and could not be used as a parameter to distinguish the two populations. It should be noted that a higher percentage of GFP-positive neurons in our cultures exhibited narrow spike waveforms in comparison to GFP-negative neurons. This could be explained by a subpopulation of interneurons which are narrow spiking. So far, narrow spikes have mainly been attributed to certain subtypes of interneurons in the mouse cortex, specifically parvalbumin positive, fast spiking interneurons (Kawaguchi and Kubota, 1993; Galarreta and Hestrin, 1999; Gibson et al., 1999; Buetfering et al., 2014). On the other hand, the presence of non fast-spiking GABAergic neurons (Markram et al., 2004) is probably also contributing to the large variance of spike waveform parameters in our study.

Although we have a reliable method to assign extracellular signals to single identified neurons in culture, we could not classify different neuronal subclasses *in vitro* based on their electrophysiological signature as suggested previously (Becchetti et al., 2012). As discussed, this electrophysiological classification is commonly used for *in vivo* recordings (Csicsvari et al., 1999; Sakata and Harris, 2009; Buetfering et al., 2014; Reyes-Puerta et al., 2014) and unambiguous cell identification has mostly been confirmed by the use of optogenetic techniques (Roux et al., 2014). But in light of the present study, special caution and further experimental studies are necessary when transferring electrophysiological classifications proven to be possible *in vivo* to different experimental models *in vitro*. This underlines the importance and strength of combined optical and electrophysiological measurements that can be easily applied for most *in vitro* preparations.

## Conflict of interest statement

The authors declare that the research was conducted in the absence of any commercial or financial relationships that could be construed as a potential conflict of interest.
